# Annotations

**Published:** 1905-01-07

**Authors:** 


### ANNOTATIONS.

### Trustees and their Obligations.

THE result of the poll of the trustees of Manchester Royal Infirmary on the question whether or not beds must be provided in a receiving house which it is proposed to build when the infirmary has been removed to Stanley Grove was declared on Thursday last week. The feature of the figures is the extraordinary proportion of abstentions. The number of voting papers issued was 1,217, and only 628, or scarcely more than half, were returned. Of these, 385 supported the resolution passed by the Board of Management, 235 were in favour of the amendment, while eight voting papers were rejected as invalid. This means a majority of 150 votes against the provision of beds, and as the trustees constitute a final court of appeal, the advocates of the adequate equipment of a receiving-house have now, we suppose, exhausted their resources. There are, we admit, arguments in favour of the policy ratified by the trustees, as well as arguments on the other side, but there is nothing to be said in favour of the course pursued by those trustees who refrained from exercising their privilege. The excuse given for their inaction is that they were, for the most part, content to leave the matter in the hands of the medical staff. But the medical staff ought not to be expected to decide issues which if the trustees are not to be mere figure-heads should rest with them. Even when the supreme question of the removal of the infirmary had to be determined, only about 800 trustees recorded their votes. No doubt the experience of Manchester is not unusual. There are trustees of many institutions who will not put themselves to the little trouble which is necessary to return their voting papers when a poll is taken. Probably they do not realise that their indifference, inattention, or want of courage to declare their opinion, may have results which they would be the first to deplore.

### The Claims of the Certificate.

THE certificate nuisance is one which is becoming increasingly familiar to the medical practitioner. Clubs, philanthropic agencies, convalescent homes, benefit societies, and last, but by no means least, educational authorities demand these documents in circumstances which practically compel the medical man to accept considerable responsibility without the slightest recognition or remuneration. The injustice of all this cannot be denied. But there is another aspect of the certificate question of social not less than of professional importance. This is the demand which is frequently or even generally made for a disclosure of the nature of the patient's ailment. Such a demand, in our opinion, ought to be steadily and consistently resisted. That an institution devoted to the reception of children or convalescents ought to take steps to protect its protégés from the risk of infectious disease every one will allow, and a negative warranty on these points is in such circumstances only fit and proper. But this is a very different position from that which exists when a child is kept away from school on medical advice. Here neither the educational authority nor any other person or organisation has the right to ask the medical man to disclose the nature or any other particulars of his patient's illness. Short of legal compulsion, such as may exist in the witness box, and in the granting of certificates either of death or under the Notification of Infectious Diseases Act, the general interest demands the cultivation of professional secrecy, and attempts to invade this ought to meet with steady and consistent opposition. There is danger, in the desire to get rid once and for all of an irritating document, of overlooking the significance of this detail of the certificate. But when attention is called to the point we are sure the profession will realise its importance. The suffering and inconvenience which would be inflicted on many poor people by a refusal to grant gratuitous certificates make medical men sympathetic towards a claim which is really a matter for general rather than individual benevolence. But that is no reason why they should provide school-attendance officers and committees on education with information which both in accord with the public interest and the traditions of the profession should be treated as private and confidential.

### Circumcision as an Aid to Identity.

THE tragedy of the Beck case has directed renewed attention to the respective values of the facts on which stress may be laid as a means of demonstrating personal identity. In the popular judgment probably no aspect of this case seemed a more decided proof of gross official blundering than the fact that of the two men so unhappily confused one had, and the other had not, been circumcised. Doubtless in this particular instance there were circumstances which rendered the mistake quite inexcusable, but with regard to the general question it may fairly be asked whether it is quite certain that the circumcised and the uncircumcised can in every individual case be readily and unquestionably distinguished the one from the other. That such a distinction can be made in most instances may be taken as certain. But is it absolute and universal, or, on the other hand, is it subject to exceptions? Probably not only the general public but the majority of medical men would not be ready to allow possible exceptions save in a very incomplete removal of the foreskin. Yet a correspondent to one of the medical journals has drawn attention to the doubtful value of the relation of the foreskin to the glans penis as an absolute proof of identity, and our own experience is in harmony with this view. There are exceptions both in one direction and the other. In the first place a lad who has been circumcised, possibly not in the most radical fashion, may certainly, in later years, be found to have a foreskin so developed that even a medical man would fail to suspect the operation. On the other hand, the habit in early life of withdrawing the foreskin behind the glans may lead to a permanent condition which, even with the greatest care, can hardly be distinguished from the result of circumcision. These facts are of all the more moment in that they conflict with the popular verdict. Even the finger imprint test does not remove the need for care in regard to other points on which identification may be based, for in some instances this test has not been applied, and on general grounds a multiplication of evidence is to be desired.

